# Charles Albert Calmette

**Published:** 1933

**Authors:** S. L. Cummins

**Affiliations:** David Davies Professor of Tuberculosis, Welsh National School of Medicine


### CHARLES ALBERT CALMETTE.

*"A PERSONAL TRIBUTE TO HIS MEMORY."*

BY

PROFESSOR S. L. CUMMINS, C.B., C.M.G., M.D.,
*David Davies Professor of Tuberculosis,*
*Welsh National School of Medicine.*

---

READERS of *The Bristol Medico-Chirurgical Journal* will have seen the numerous obituary notices of the late Dr. Charles Albert Calmette which have appeared in the Press. It is, therefore, unnecessary to refer at length to his younger days spent as a naval surgeon in Chinese waters or to his first entry into independent scientific study as a young bacteriologist picked out by Pasteur for special work at Saigon. It was there that he made his earliest contributions to preventive medicine and obtained world-wide renown for his researches on snake venom and their treatment by specific anti-sera. What may, perhaps, be of some interest is a personal account of a charming French gentleman, who was also one of the greatest medical scientists of our time.

My first encounter with Calmette was in the town of Lille the week after the Germans had been driven from it in the autumn of 1918. Having recently taken over the duties of Adviser in Pathology to the British Forces in France from Sir William Leishman, it was my duty to keep in touch with the mobile laboratories as our troops pushed forward; and so I hurried up to Lille partly to enjoy the opportunity of meeting Calmette, already well-known to me by reputation, and also to ask for his help in case any of our laboratory units were in his neighbourhood. I found, however, that Major Fitzgerald, of the Canadian Army Medical Corps (now the Director of the Connaught Laboratories at Toronto), had already, on his own initiative and with Calmette's kind consent, installed his mobile laboratory in the Pasteur Institute at Lille, and had received every possible courtesy and help from its distinguished Director. It was wonderful to meet this renowned scientist, still at his post in spite of the German invasion, and now once more amongst friends and Allies at the end of his long ordeal.

Where a pathologist of world-wide reputation, controlling an institution of international value, might have expected nothing but courtesy and co-operation even from a foe, Calmette had been exposed to many indignities and hardships even by men whom he had known as colleagues in pre-war days. His beloved wife had actually been taken away into Germany as a hostage, where she was kept for months incarcerated with a number of other detained women, some of undesirable character, for no crime at all, except that of being a French lady of distinction. Yet, in spite of all these things, there was no inveterate rancour or bitterness in the heart of Calmette; and he exhibited with gaiety as well as pride the last rabbit which he had been permitted to preserve for the maintenance of the rabies virus.

Our next encounter was in Paris, after he had assumed the post of Assistant Director of the Pasteur Institute under Dr. Roux. He was no longer the tired leader of a forlorn hope, as he had seemed at Lille, but now he stood resplendent in the smart uniform of a Surgeon-General in the French

Army. His kindness and his affability, however, were in no way diminished. No holder of a distinguished position was ever more approachable, helpful and kind. At the Conference of the International Red Cross Society, held at Cannes in the Spring of 1919, Calmette was present as one of the Mission of French Scientists delegated to attend. It was a time when the attitude of many of us towards our late enemies was still embittered; and yet Calmette, who had suffered deeper personal trials than any, showed a real sense of the importance of re-establishing the bonds that should unite doctors across national frontiers in the world-wide conflict with disease. When, at a later date, it was my privilege to be appointed Professor of Tuberculosis in the University of Wales, I had frequent occasions to maintain personal touch with Dr. Calmette. It was then that I came to recognize fully the unequalled value of this great specialist in relation to tuberculosis.

Perhaps it may be permissible to speak a few words on this subject.

The contributions of Calmette to the study of tuberculosis and to its prevention have been on a scale unequalled by any individual worker since Robert Koch. Villemin, an army doctor, had been the first to demonstrate clearly the transmissibility of tuberculosis from man to animals and from one animal to another, thus proving its infective nature; and Calmette, a naval medical officer, was amongst the pioneers in following up this problem of infectivity, with a view to its prevention on scientific lines. The first objective was clearly to map out the distribution of infection, as opposed to disease, in human aggregations. Simultaneously with Wolff-Eisner, and independently of him, Calmette published in June, 1907, the first account of an ophthalmo-reaction, demonstrating that the application of diluted tuberculin to the conjunctiva could elicit, in sensitive persons, a marked inflammatory response of a specific though transitory kind. This discovery, published within a month of Von Pirquet's first announcement of his cutaneous test, was destined to be widely used for a short period, but it proved dangerous in certain cases, and Calmette himself was amongst the first to advocate the safer Von Pirquet technique as the best method for working out the distribution of infection in different racial groups and at varying age periods. He did not, however, stop short at the mere publication of a good method. Instead, he threw himself into a laborious propaganda for the use of cutaneous tuberculin tests in the native populations throughout the French Colonies, and with the help of the Pasteur Institutes in the Colonies was instrumental in accumulating a vast mass of evidence as to the state of tuberculization of the natives of French Africa, Indo-China and other French Dependencies from 1911 onwards.

Still another contribution was already under way. During the years of trial at Lille Calmette had not been idle. It was during those years that he built up from existing literature the wonderful book which has, perhaps, done more than any other publication to spread through the world scientific conceptions of tuberculosis. The first publications of "L'Infection Bacillaire et la Tuberculose chez l'Homme et chez les Animaux" appeared in 1919. His object in writing it was, in his words, "to gather together from the most important works on tuberculosis since the publication of Straus's book in 1895," and from his personal researches, "the scientific principles on which, in the actual state of our knowledge, should be based the fight against the most terrible infectious disease from which humanity suffers." It is impossible to contemplate this book without admiration for the industry, accuracy and determination required to bring it to fruition. Its reception by the medical profession is a good index of its worth. The first (1919) and second (1922) editions were rapidly sold out, and the third (1927), a still more comprehensive work, is always in great demand amongst students of tuberculosis.

Finally came his greatest experiment, the inception of an attempt at tuberculosis prevention on lines peculiarly suitable to a follower of Pasteur—the use of a living attenuated virus, B.C.G., as a vaccine against tuberculosis. With his colleague Guèrin Calmette had worked for sixteen years at the attenuation of a bovine strain of tubercle bacillus handed him at its full virulence by the veterinary bacteriologist Vallée. Calmette had formed the opinion that ox bile, while not interfering with the growth of tubercle bacilli, might possess the property of reducing their virulence by its physical action on the fatty and waxy constituents of cultures, and it was through successive cultivations of this strain on bile-potato medium that attenuation was at last successfully brought about, until a stage was reached when the virulence for laboratory animals had diminished almost to vanishing point. It was now safe to attempt experiments on human beings, and his trusted colleagues, Weill-Hallé and Turpin, took the next step—the feeding of this vaccine to infants in their earliest days, at a time when the permeability of the intestine was at its maximum and the tissues not yet hypersensitive through infection. The earliest vaccinations were carried out between 1921 and 1924, and it was soon clear that the vaccine was innocuous in the doses used. Since that time the use of B.C.G. has grown to enormous proportions throughout France and her Colonies, as well as in many parts of Europe and America. England alone has held aloof.

In 1927, the Medical Research Council gave close attention to the possibility of a controlled test on children in this country, and it was my lot to be sent to Paris to discuss the question with Professor Calmette and report. As a result of this visit a programme was formulated, based on the use of an adequate amount of vaccine supplied by the Pasteur Institute, to be given to selected children living in contact with " open " cases, under conditions where close supervision might be carried out during subsequent months and years. With characteristic courtesy, Calmette at once promised to supply all the vaccine required free of charge.

The statistics quoted in France had been subjected, however, by Professor Major Greenwood and others to rather damaging criticism, not with regard to the innocuity of the vaccine, which was admitted, but as to its utility in the doses used and when given by the mouth. Under these circumstances, the Medical Research Council decided that no official test on children was desirable until further experiments on monkeys and other animals had been carried out for the Council by Dr. Stanley Griffith. This delay was a great disappointment to Calmette. Writing to me in 1930, he could not refrain from a sly dig at the Labour Government then in office ! " Je sais que votre Gouvernement, malgré son étiquette travailliste, est le plus conservateur qui soit au monde, mais il me semble tout de même qu'il finira par comprendre qu'il est de son intérêt de ne pas se priver d'un moyen efficace et inoffensif de combattre la tuberculose."*

In a postcript to the same letter he refers amusingly to a much advertised non-medical producer of vaccines and serums against tuberculosis in the neighbouring country of

* " I know that your Government, in spite of its Labour label, is the most conservative in the world, but it seems to me all the same that it will realize in the long run that it is to its interest not to deprive itself of an effective and innocuous weapon for combating Tuberculosis."

Switzerland, " qui parait trouver, auprès des autorités officielles britanniques, une certaine complicité et une bienveillance qu'il nous serait agréable de voir accorder aussi à nos travaux ! "*

The Lübeck disaster came as a terrible set-back to the use of B.C.G. throughout the world; but the attitude of some scientists amongst our late enemies was a great comfort and support to Calmette, and he sent me, with obvious pleasure, a copy of a noble letter which he had received from Dr. E. Altstaedt, one of those convicted at the trial at Lübeck, written in most generous terms, entirely exonerating Calmette from any responsibility for the disaster and finishing in the following words :—

> " The judgement of Lübeck is pleasant at least in the sense that it has resulted in the official proof of a contamination."

The Lübeck tragedy and the subsequent trial had involved mental anguish to Calmette, and it is doubtful if his bodily health ever quite recovered from the strain. Even the final judgement was insufficient to remove the impression of danger which the disaster had fostered in the minds of some of those who might have contemplated the use of the vaccine, and its world-wide use has undoubtedly become limited as a result.

It is, perhaps, still too early to decide whether the claims for B.C.G. are to be accepted in full, but its innocuity is proved; and the recent work of Dr. A. Stanley Griffith and Professor J. B. Buxton at Cambridge has demonstrated beyond question that by its means calves can be given an almost complete protection against large doses of virulent bovine tubercle bacilli.

It is quite certain that the vast experiment undertaken by Calmette represents one of the most important, as well as the most courageous, investigations ever initiated into the problem of prevention of tuberculosis. Whether it may prove as successful as Calmette had hoped, or whether other inoculation methods, safer and more efficient, may be found in the future, the vast B.C.G. experiment must necessarily result in a great increase of our knowledge about the responses of the body to infection with tubercle bacilli.

In the death of Calmette medical science has lost one

* " Who appears to find among British Government Officials a sort of complicity and goodwill which it would be very pleasing to see extended to our own work."

of its foremost investigators. To those who had the privilege of his friendship the loss is still deeper. Humanity has no more delightful representative than a cultured, witty and intelligent Frenchman. All these qualities were exhibited in their finest form in Charles Albert Calmette.

### Meetings of Societies.

